# Timestamp Estimation in P802.15.4z Amendment †

**DOI:** 10.3390/s20185422

**Published:** 2020-09-22

**Authors:** Ioan Domuta, Tudor Petru Palade, Emanuel Puschita, Andra Pastrav

**Affiliations:** Communication Department, Technical University of Cluj-Napoca, 400027 Cluj-Napoca, Romania; tudor.palade@com.utcluj.ro (T.P.P.); Emanuel.Puschita@com.utcluj.ro (E.P.); Andra.PASTRAV@com.utcluj.ro (A.P.)

**Keywords:** UWB, 802.15.4z, timestamp detection, ranging, multipath, frequency fading

## Abstract

Due to the known issue that the ranging in the 802.15.4™-2015 standard is prone to external attacks, the enhanced impulse radio (EiR), a new amendment still under development, advances the secure ranging protocol by encryption of physical layer (PHY) timestamp sequence using the AES-128 encryption algorithm. This new amendment brings many changes and enhancements which affect the impulse-radio ultra-wideband (IR-UWB) ranging procedures. The timestamp detection is the base factor in the accuracy of range estimation and inherently in the localization precision. This paper analyses the key parts of PHY which have a great contribution in timestamp estimation precision, particularly: UWB pulse, channel sounding and timestamp estimation using ciphered sequence and frequency selective fading. Unlike EiR, where the UWB pulse is defined in the time domain, in this article, the UWB pulse is synthesized from the power spectral density mask, and it is shown that the use of the entire allocated spectrum results in a decrease in risetime, an increase in pulse amplitude, and an attenuation of lateral lobes. The paper proposes a random spreading of the scrambled timestamp sequence (STS), resulting in an improvement in timestamp estimation by the attenuation lateral lobes of the correlation. The timestamp estimation in the noisy channels with non-line-of-sight and multipath propagation is achieved by cross-correlation of the received STS with the locally generated replica of STS. The propagation in the UWB channel with frequency selective fading results in small errors in the timestamp detection.

## 1. Introduction

The wireless localization is a key part of many emerging technologies: internet of things (IoT), intelligent transportation systems (ITS), autonomous robots, or unmanned aerial vehicles. For many critical applications, localization accuracy is a basic requirement of localization systems. Due to its large bandwidth, the impulse radio ultra-wideband (IR-UWB) technology provides the best precision in range measurement by time-of-flight (ToF) estimation.

The basic feature in the accuracy of estimating the ToF is the shape of the pulse, more precisely the speed of increase of the pulse front. Nowadays the IEEE Task Group 4z, (TG4z), [1] is working on the enhanced impulse radio (EiR) project focused on localization safety improvement. This new amendment proposes a new UWB reference pulse and a time domain mask. In this paper, the UWB pulse is synthesized from power spectral density (psd) specified by the 802.15.4-2015 standard [2]. Two shapes of pulses were synthesized and compared, the first pulse being synthesized using only the central lobe of the psd mask and the second one being synthesized from the entire allocated spectrum.

The EiR amendment proposes that the timestamp estimation is validated by the cross-correlation of locally generated STS replica with received STS sequence. To avoid the interferences, the pulses are spread out on a symbol. This article proposes a supplementary spreading by a bit position modulation with a randomly generated sequence and shows, by simulated experiments, that lateral lobes of cross-correlation are mitigated by this modulation.

The behavior of the proposed methods is analyzed in a noisy radio channel with non line-of-sight (NLOS) and multipath propagation. The channel impulse response is estimated and subsequently used for the generation of a local replica of STS.

The main contributions of this article are that it:

- demonstrates that the inclusion of lateral lobes with very low power spectral density (−51.3 dBm and −59.3 dBm) in pulse synthesis leads to a pulse with a tighter shape and a steeper rising edge than the pulse recommended by EiR;
- shows that, by a random spreading of STS, that it results in easier extraction of the main lobe by mitigating lateral lobes of cross-correlation.

This paper is organized as follows. Section 2 presents state of the art research in the field. In Section 3, the UWB pulses are synthesized. Section 4 presents the random spreading of STS sequence and timestamp estimation in noisy channels and NLOS propagation. All sections incorporate simulated experiments, and because the simulation results from a subsection are used in the subsequent ones, they will be presented along with the theoretical aspects in the corresponding subsection.

## 2. Literature Review

The UWB radio holds a large bandwidth, but the harmonized standards [3] impose upper limits for power spectral density (−41.3 dBm/MHz, which is under the noise floor), resulting in great difficulty in the extraction of signal from noise. The research in [4,5] shows the presence of intra-symbol interference (IASI), inter-symbol interference (ISI), and multipath interference (MUI). In order to minimize the interferences, the pulse should have a small duration of the leading lobe and a high attenuation of the side lobes. It must be noted that this small duration of the main lobe can lead to an excess in bandwidth. The pulse shape has to be a compromise between the regulatory compliance, the need of low voltage and low power supply, low duration for maximization of data rate, ranging accuracy, and minimalization of interferences. In most cases, the UWB pulse is synthesized from Gaussian impulse [6], its derivatives [7], or a linear combination of Gaussian pulses [8]. Keshavarz et al. [9] infer the weights of the derivatives in the impulse structure by particle swarm optimization (PSO) algorithm, and PSO algorithm is used for optimization of the architecture of a UWB transmitter [10]. A linear combination of Gaussian monocycles with weight optimization by semidefinite programming is used for pulse synthesis [11]. Baranauskas and Zelenin present a direct waveform synthesis of UWB pulse by high speed DAC [12].

The EiR amendment specifies two pulses [13] as boundaries for the UWB pulse and a time domain mask [14] as a constraint for pulse shape. The UWB pulse synthesized from the entire allocated spectrum falls into the time domain mask, has high energy, and can be used as a reference in UWB pulse design.

In the 802.15.4 standard [2], the UWB PHY is specified in detail and, the transceivers manufactured in this technology are widely used in localization. However, several researches show that the range measurement in the current technology is prone to external attacks. Francillon et al. [15] present a relay attack, Taponecco et al. [16] show a delay attack and Singh et al. [17] propose a modulation scheme that secures the distance measurement against relay attack. The EiR project [1] brings a lot of improvements, including UWB reference pulse shape, preamble symbols revision, addition of scrambled timestamp sequence for secure ranging, an increase in data rate and PHY payload length, and the modification and addition of a new MAC primitive for key management. An overview of the EiR standard is presented in the work of Sedlacek et al. [18].

This article is limited to estimating the timestamp, without going into detail regarding ranging or location methods. Alarifi et al. perform a deep analysis of ultrawide band indoor positioning Technologies [19]. Several works deal with the wireless localization in internet of things (IoT) [20,21,22] and many research depict the localization in vehicular technologies [23,24,25].

## 3. UWB Pulse Synthesis

The pulse shape plays an essential role in the ranging accuracy and in reaching the maximum distance, while maintaining regulatory compliance. The 802.15.4-2015 standard, hereinafter called ‘old standard’ has been defined as a root raised cosine reference pulse. As this pulse has a precursor, it can mask the attenuated first path signal. The TG4 proposes [13] that the transmitted pulse shape p(t) to be constrained by the time domain mask, specified by the standard. The EiR specifies that the pulse risetime, 10–90%, for 500 MHz channels has to be maximum 2 ns.

**Figure 1:**
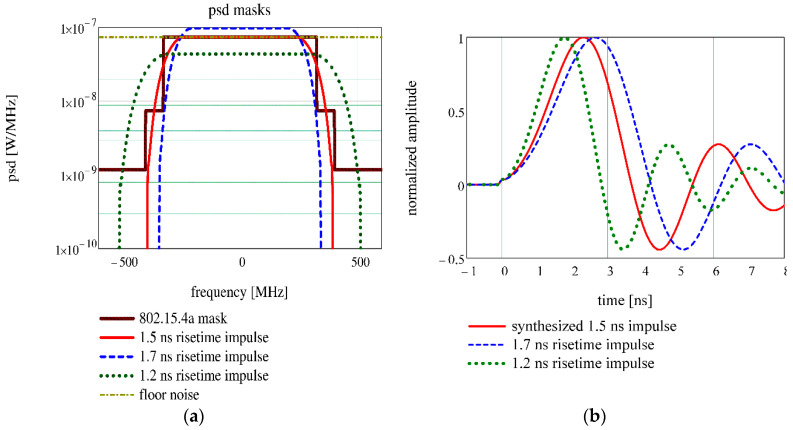
UWB impulse synthesis: (**a**) Spectral masks; (**b**) The pulses in time domain.

This paper proposes the synthesis of UWB pulse from the compliant power spectral density mask (psd) [2] by Kolmogorov factorization (detailed in Appendix A) [26], because this method provides a minimum phase pulse, as it is specified by the EiR. The old standard specifies the psd mask as it is depicted in Figure 1a, namely the trace 802.15.4a mask. The power is expressed in Watts, in order to get the pulse amplitude in volts (1 Ω load). For pulse synthesis, a new spectral mask is designed, trace 1.5 ns risetime impulse, using a raised cosine profile

(1) $$H=\{\begin{array}{cc} psd_{H} & \left|f-f_{c}\right|<\left(1-\beta \right)F_{c}\;\\ \left(\frac{psd_{H}+psd_{L}}{1}\right)\left(1+\cos\left[\frac{\pi }{2\beta }\left(\frac{f-f_{c}}{F_{c}}-1+\beta \right)\right]\right)+psd_{L} & \left(1-\beta \right)F_{c}\leq \left|f-f_{c}\right|\leq \left(1-\beta \right)F_{c}\\ 0 & otherwise \end{array}$$

where central frequency $f_{c}=0$, cutoff frequency $F_{c}=315\;\mathrm{MHz}$, roll-off factor β=0.25 and $psd_{H}\;and\;psd_{L}$ are the high and low value of psd mask. The sampling frequency is $f_{s}=10\;\mathrm{GHz}$ and the window length is $N=10^{4}$ samples for 1 MHz frequency resolution.

Different from standard [3], where the pulse energy is averaged on a 1 ms interval, in the paper, the mean power is computed on a 1 μs interval, considering that the pulse repetition frequency PRF=1 MHz. Accordingly, the pulse amplitude is inferred based on this PRF. The EiR defines many mean PRFs and, in order to comply with the regulations, the determined amplitude has to be scaled with $\sqrt{PRF\;\left[\mathrm{MHz}\right]}$. Furthermore, the regulation imposes the pulse peak power to a value that shall not exceed 0 dBm on 50 MHz bandwidth, and the impulse has to respect this restriction too.

Figure 1b shows the synthesized pulse, trace synthesized 1.5 ns impulse, compared to EiR compliant pulses for the 499.2 MHz bandwidth (i.e., 1.7 ns risetime impulse and 1.2 ns risetime impulse traces). The synthesized pulse is situated between recommended pulse limits, so it respects the specifications. The traces 1.7 ns risetime and 1.2 ns risetime in Figure 1a show that the pulses suggested by the EiR do not fit exactly in the standard psd mask, the first exceeding the maximum psd and the second exceeding the bandwidth.

**Figure 2:**
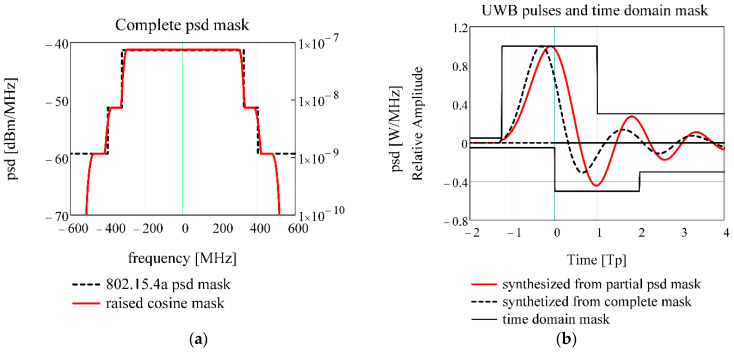
UWB impulse synthesis from complete spectral mask: (**a**) Spectral mask from multiple raised cosine profiles; (**b**) Time domain mask and synthesized pulses.

Recently, TG4z has defined a time domain mask for UWB impulse [12], as illustrated in Figure 2b. Based on this mask, it is appropriate to search for a new UWB pulse shape which falls in this mask for impulse energy maximization and risetime reduction. The Cramer–Rao lower bound in ToF estimation is inversely proportional to effective bandwidth [27], so it is convenient to use the lateral lobes of low power for pulse synthesis. In order to do this, a new psd mask is designed using a sum of raised cosine profiles, Figure 2, having the following parameters: $\left(F_{c}{}_{1}=315\;\mathrm{MHz},\;\beta_{1}=0.05\right);\;\left(F_{c}{}_{2}=400\;\mathrm{MHz},\;\beta_{2}=0.05\right);\;\left(F_{c}{}_{3}=500\;\mathrm{MHz},\;\beta_{3}=0.05\right)$.

Figure 2b shows that the pulse synthesized from complete mask has a smaller risetime and a stronger attenuation of lateral lobes compared to the pulse synthesized from partial psd mask. As such, the pulse synthesized from complete mask allows for more precise timestamp estimation and less interferences. The pulse synthesized from complete mask has the amplitude 0.187 V/1 Ω/1 μs, higher energy, low risetime $T_{10\%\to 90\%}=1.2\;\mathrm{ns}$ and smaller lateral lobes than the pulse synthesized from partial psd mask which has an amplitude of 0.164 V/1 Ω/1 μs and a risetime of $T_{10\%\to 90\%}=1.5\;\mathrm{ns}$. The pulse risetime plays a key role in timestamp estimation precision. Therefore, it is preferable to choose the pulse synthesized from complete mask as UWB reference pulse.

The degree of spectrum usage is evaluated by normalized effective signal power (NESP) [6]

(2) $$\mathrm{NESP}=\frac{\int {\left|S\left(f\right)\right|}^{2}df}{\int M\left(f\right)df}$$

where ${\left|S\left(f\right)\right|}^{2}$ is spectral density of the impulse and M(f) is allocated spectral mask.

Because the signal does not have a uniform distribution on the entire spectrum, the effective bandwidth β is defined

(3) $$\beta =\sqrt{\frac{\int f^{2}{\left|S\left(f\right)\right|}^{2}df}{\int {\left|S\left(f\right)\right|}^{2}df}}$$

Usually, the quality of the UWB pulse synthesis is defined in terms of occupied bandwidth and pulse duration. Table 1 compares the quality parameters of proposed pulse with P802.15.4z reference pulse.

The minimum uncertainty, σ, for range estimation in the time of arrival method is quantified by Cramér–Rao lower bound (CRLB) [28]

(4) $$\sigma =\frac{c}{\beta \sqrt{8\pi^{2}SNR}}$$

where β is effective bandwidth, c is speed of the light, and SNR is signal to noise ratio.

Relation (100) clearly shows that for minimizing the uncertainty the effective bandwidth has to be maximized, but within the limit of regulations.

## 4. Timestamp Estimation in the 802.15.4z Standard

### 4.1. Timestamp Estimation by Random Spreading of STS

The new standard brings in a new physical protocol data unit (PPDU) structure by incorporating the STS for secure ranging. The STS is encrypted using the AES-128 algorithm, the time of arrival estimation is achieved on STS, and the range measurement is validated only if the received STS cross correlated with the locally generated reference exceeds the “match level” [29] threshold.

**Figure 3:**
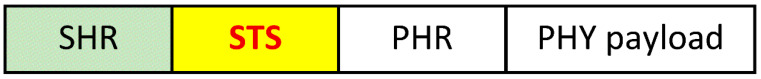
PHY frame format in the 802.15.4z amendment.

The default PHY frame format proposed by EiR [29] is depicted in Figure 3, where SHR is the synchronization header (preamble), STS is the scrambled timestamp sequence, and PHR is the PHY header.

To avoid the inter-pulse interferences, [30] stipulates that every component $B_{k}$ of Ipatov ternary symbol (ITS), or STS, is spread out on a symbol of length $\delta_{L}$ by $\sum_{n=0}^{\delta_{L}-1}\delta \left(n\right)\;p\left(t-nT_{ch}\right)$; where δ(n) is Kronecker delta, p(t) is UWB pulse and $T_{ch}=2\;ns$ is chip duration. To mitigate the side lobes of correlation, this paper proposes a supplementary spreading by a randomly generated sequence $S_{k}$. The sequence of length N is:

(5) $$s\left(t\right)=\sum_{k=0}^{N-1}B_{k}\sum_{n=0}^{\delta_{L}-1}\left(\delta \left(n\right)\cdot p\left(t-\left(k\cdot \delta_{L}+S_{k}n_{s}+n\right)T_{ch}\right)\right).$$

**Figure 4:**
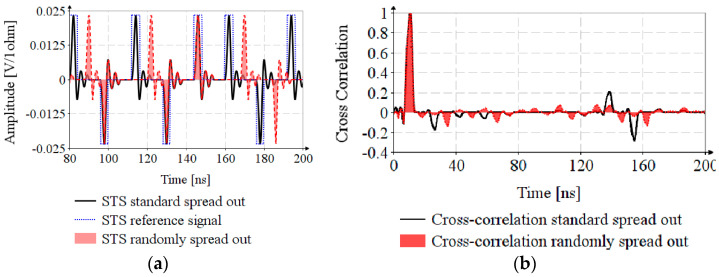
Timestamp estimation by STS: (**a**) UWB pulse position in STS sequence; (**b**) cross-correlation of STS sequences with reference signal.

The STS sequence of length N=128, *s*(*t*), is generated by taking $B_{k}=1-2A_{k}$, A being the result of AES encryption. The peak pulse repetition frequency (PRF) is 499.2 MHz, the mean PRF is 62.4 MHz, resulting $\delta_{L}=8\;\mathrm{chips}$. Figure 4a depicts the STS sequences: STS standard spread out is generated according to the standard specifications, $S_{k}=0$, STS reference signal is the signal needed to achieve correlation and for STS randomly spread out, the $S_{k}$ is generated based on a linear feedback shift register with the characteristic polynomial $x^{3}+x+1$. The cross-correlation of the reference signal and STSs are shown in Figure 4b. The ratio between the main lobe and maximum side lobe is η≅10 for cross-correlation randomly spread out and η≅3.33 for cross-correlation standard spread out, which proves the efficiency of random spreading.

Figure 4a shows that the random spreading increases the risk of IASI. Therefore, it is necessary to perform the analysis of timestamp estimation for propagation in noisy channel and for multipath propagation.

### 4.2. Sounding the Channel with Multipath Propagation

The channel sounding is the estimation of the channel impulse response (CIR) using preamble sequence, in order to remove the noise and design the channel equalizer.

The frequency-dependent path gain G(d,f) is modeled considering an isotropic radiation pattern, with the “antenna attenuation factor” [31] of ½:

(6) $$G\left(d,f\right)=\frac{P_{Rx}\left(d,f\right)}{P_{Tx}\left(f\right)}=\frac{1}{2}\;G_{0}\eta_{Tx}\eta_{Rx}\frac{{\left(f/f_{c}\right)}^{-2\left(\kappa +1\right)}}{{\left(d/d_{0}\right)}^{n}}\;,$$

where $P_{Rx}\left(d,f\right),P_{Tx}\left(f\right)$ are received and transmitted power, $\eta_{Tx},\eta_{Rx}$ are the transmission and reception antenna gains, $G_{0}$ is the path gain at reference distance $d_{0}$, d is the distance between transmitter and receiver, n is the path gain exponent, $f_{c}$ is the carrier frequency, f is the frequency and κ is the frequency decaying factor.

The frequency decaying factor follows the Friis equation and, in Equation (6), it has the value κ=0. The path loss varies from n=1.2 in industrial LOS, n=1.76 in outdoor LOS, to n=4.58 in residential NLOS. It is noted that multipath propagation in the industrial environment leads to an increase in the path gain.

Using the Saleh-Valenzuela statistical model, the propagation paths are designed as the sum of clusters, every cluster having multiple rays. The impulse response $h^{m}\left(t\right)$ is

(7) $$h^{m}\left(t\right)=\sum_{l=0}^{L}\sum_{r=0}^{K}a_{r,l}exp\left(j\emptyset_{r,l}\right)\delta \left(t-T_{l}-\tau_{r,l}\right),$$

where $a_{r,l}$ is the tap weight of r ray in cluster l, $\emptyset_{r,l}$ is the ray phase, $T_{l}$ is the delay of l cluster and $\tau_{r,l}$ is the delay of r ray relative to cluster l front. The intervals between the time arrivals of the clusters is modeled as Poisson process with the arrival rate Λ, and the ray delays inside the cluster are modeled as a mixture of two Poisson processes with arrival rates $\lambda_{1},\;\lambda_{2}$ and mixing weight β. The mean power of arriving clusters follows an exponential decay with time constant Γ, having a normal distribution around the mean value $\sigma_{cluster}$, and the cluster shape also bears to an exponential decay with time constant γ.

In the above CIR model, the first path has the highest energy. In non-line-of-sight propagation, there are cases when the first path is strongly attenuated. For such situations, [31] proposes a new modeling for the first path.

(8) $$E\left\{a_{k,0}\right\}\propto \left(1-\chi exp\left(-\frac{\tau_{k,l}}{\gamma_{rise}}\right)\right)exp\left(-\frac{\tau_{k,l}}{\gamma_{1}}\right),$$

where χ describes the attenuation of the first path, $\gamma_{rise}$ determines how fast the power delay profile (PDP) increases, and $\gamma_{1}$ determines the profile decay. By joining Equations (7) and (8), the CIR, *h*(*t*) can be found.

The preamble sequence consists of a string of 32 or 64 Ipatov ternary symbols, every symbol having 91 elements with 81 non-zero elements [30]. An Ipatov symbol has “perfect” periodic autocorrelation, i.e., all side lobes of autocorrelation are zero, and using Wiener-Hopf equation, by cross-correlation of the received signal y(t) with the input Ipatov sequence I(t) results immediately the CIR, h(t)

(9) $$y\left(t\right)⋆I\left(t\right)=h\left(t\right)*I\left(t\right)⋆I\left(t\right)=h\left(t\right)*R_{I,I}\left(t\right),$$

where autocorrelation of ITS is: $R_{I,I}\left(0\right)=N;R_{I,I}\left(t\right)=0\;\mathrm{for}\;t\neq 0$.

For outdoor, NLOS and multipath propagation environment, the channel PDP, h(t), is modeled based on Equations (7) and (8), with the parameters retrieved from [31], and synthesized in Table 2.

**Figure 5:**
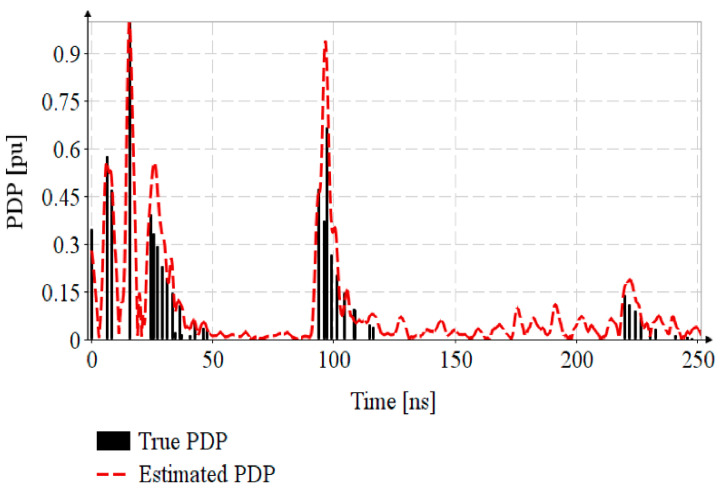
CIR estimation for channel with multipath propagation.

For channel sounding, the EiR standard specifies a mandatory preamble sequence of 32 or 64 ITSs, the Ipatov symbol having a number of 91 elements with 10 zero elements. In this paper, the CIR is estimated only using one ITS with a total of 57 elements including 8 zero elements [32]. The transmitted sequence of pulses, i(t), is generated based on Equation (5), with $B_{k}=I_{k}$, where $I_{k}$ is the *k*th element of ITS. The received signal $y_{i}\left(t\right)=h\left(t\right)*i\left(t\right)$ is the convolution of PDP with the emitted sequence. The CIR estimation, $\hat{h\left(t\right)}$, is achieved by the cross-correlation of the received signal y(t) with the Ipatov symbol I. Figure 5 shows that the CIR estimation, estimated PDP, is close enough to the true PDP. By successive transmission of ITSs contained in the preamble, the estimations are accumulated and averaged, resulting in SNR reduction and CIR estimation improvement.

This CIR estimate will be used in the following paragraphs, for analysis of timestamp estimation in channels with multipath propagation. This is a classical channel model, more detailed models are being published in the recent research [33,34].

### 4.3. Timestamp Estimation in Channel with NLOS and Multipath Propagation

The EiR specifies that STS consists of 32, 64, 128 AES-128 sequences, successively transmitted, but in this section only one AES sequence is considered for analysis the impact of multipath propagation.

The STS is generated based on Equation (5) with random spread out, and for propagation simulation it is convoluted with h(t).

In the usual way, the received signal is passed through an equalizer filter and cross-corelated with the STS reference $s_{r}\left(t\right)$. In this article, an easier way for timestamp detection is proposed, that is, to generate a virtual propagated STS reference by convolution of the STS reference with the estimated CIR, $\hat{h\left(t\right)}$, and cross-correlation of received sequence $y_{s}\left(t\right)$ with locally generated replicas $\hat{y_{r}\left(t\right)}$ as depicted in Algorithm 1.
**Algorithm 1**: Timestamp estimation in multipath propagation**Inputs**: STS reference (AES-128 sequence), $s_{r}\left(t\right)$; Received STS, $y_{s}\left(t\right)$ 1. Generate locally replica of STS:$\hat{y_{r}\left(t\right)}=\hat{h\left(t\right)}*s_{r}\left(t\right)$
 2. Compute the received sequence:$y_{s}\left(t\right)=h\left(t\right)*s\left(t\right)$ 3. Timestamp estimation:$r_{rs}\left(t\right)=\hat{y_{r}\left(t\right)}⋆y_{s}\left(t\right)$**Output**: return
$r_{rs}\left(t\right)$

**Figure 6:**
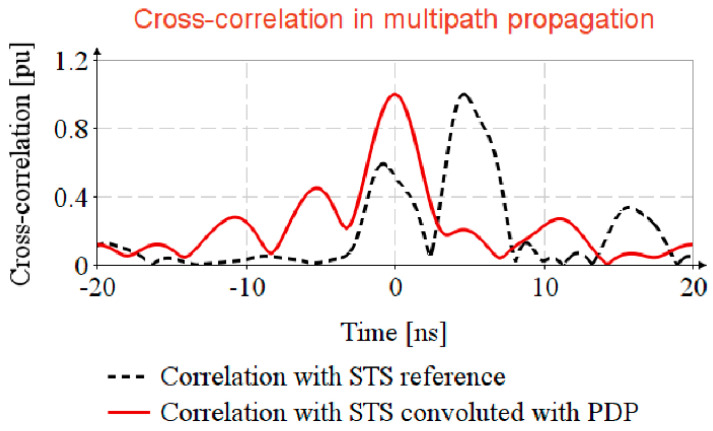
Timestamp estimation in channel with NLOS and multipath propagation.

Figure 6 shows that by cross-correlating the received STS, $y_{s}\left(t\right)$, with the STS reference, $s_{r}\left(t\right)$, the timestamp is not detectable, (see the correlation with STS reference trace), and that the cross-correlation of received signal with the locally generated replica, $\hat{y_{r}\left(t\right)}$, the side lobes are strongly attenuated (see the correlation with STS reference convoluted with PDP trace).

### 4.4. Timestamp Estimation in Noisy Channel with NLOS and Multipath Propagation

The EiR standard specifies that the typical range of radio is 100 m. For timestamp estimation in noisy channel we consider that the transmitter is situated at 22 m and the noise source is situated at reference distance of 1 m.

**Figure 7:**
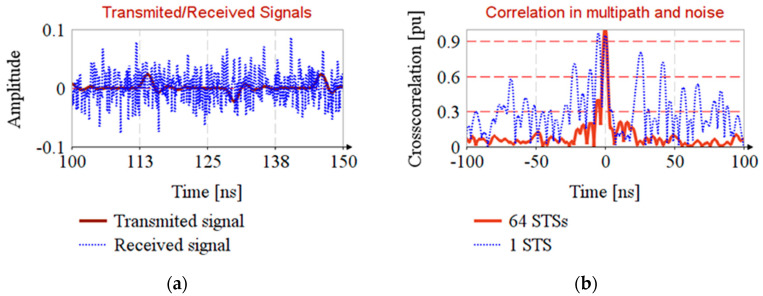
Timestamp estimation in noisy channel with multipath propagation.

The transmitter emits with maximum compliant power (Figure 2a) and the noise source emits with floor noise level (i.e., −41 dBm/MHz) [35]. In order to mitigate the effect of noise, consider that the receiver has a 1 GHz passband filter on the input. The standard deviation of noise is σ=0.0228 V and the signal to noise ratio on the receiver is SNR=−38 dB. In this situation, the timestamp is undetectable from only a single AES-128 sequence (Figure 7b, trace 1 STS), so STS will consist of multiple AES sequences as the EiR standard specifies.

The timestamp detection is detailed in Algorithm 2 and the results are shown in Figure 7.
**Algorithm 2**: Timestamp estimation in noisy channel **Inputs**: STS reference (AES-128 sequence), $s_{r}\left(t\right)$; Received STSs, $y_{s}^{i}\left(t\right);$ Number of STS, N; 1. Generate locally replica of STS:$\hat{y_{r}\left(t\right)}=\hat{h\left(t\right)}*s_{r}\left(t\right)$
 2. Compute the received sequences:for i∈[0,N−1]  do:     
$y_{s}^{i}\left(t\right)=h\left(t\right)*s\left(t\right)+n^{i}\left(t\right)$
 3. Average the received sequences:$y_{s}\left(t\right)=\frac{1}{N}\overset{N-1}{\underset{i=0}{\sum }}y_{s}^{i}\left(t\right)$
 4. Timestamp estimation:$r_{rs}\left(t\right)=\hat{y_{r}\left(t\right)}⋆y_{s}\left(t\right)$
 Where $n^{i}\left(t\right)$ is the noise with standard deviation σ**Output**: return $r_{rs}\left(t\right)$

### 4.5. UWB Channel with Frequency Selective Fading

**Figure 8:**
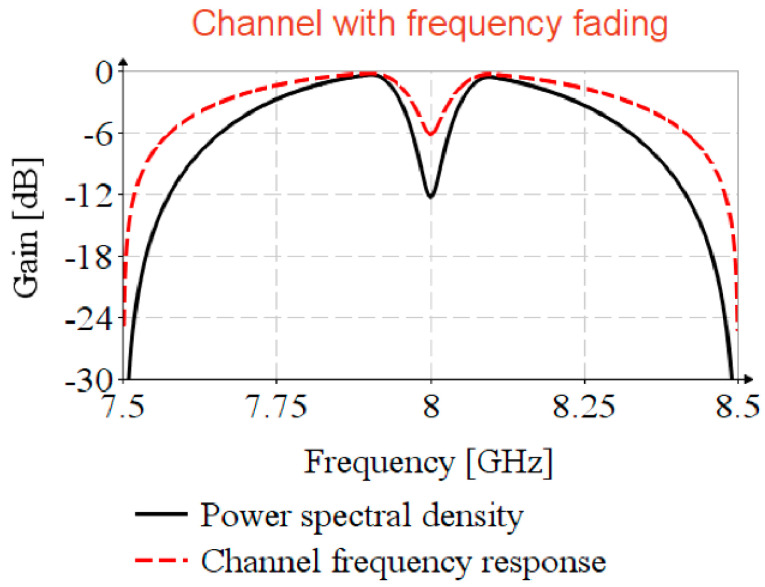
Psd mask and channel response.

Depending on antenna design, or antenna position relative to external objects, it is possible to encounter frequency selective fading [35,36]. To analyze the impact of frequency fading in timestamp detection, consider UWB channel 9 with the spectral mask profile power spectral density, illustrated in Figure 8, having a selective fading of 12 dBm at carrier frequency and frequency decaying factor of κ=0.13. The channel frequency response, H(f), is achieved by Kolmogorov factorization (Appendix A).

To estimate the CIR, $\hat{H\left(f\right)}$, the Ipatov symbol, i(t), is shifted on to the carrier, $f_{c}$, by complex modulation with $\exp(j2\pi f_{c}t$). The cross-correlation is performed in the frequency domain, and to avoid the circular correlation, the series is padded with zeros. The propagation of STS in the faded channel is simulated by the shifting of the STS on to the carrier and by the convolution with H(f). The timestamp estimation is detailed in Algorithm 3.
**Algorithm 3**: Timestamp estimation in channel with frequency selective fading**Inputs**: Ipatov symbol, i(t); STS, s(t);   **CIR estimation:** 1. Lift up the Ipatov symbol on the carrier:$i_{c}\left(t\right)=e^{j2\pi f_{c}t}\cdot i\left(t\right)$ 2. Complete the series with zero and perform FFT.$I_{c}\left(f\right)=FFT\left(i_{c}\left(t\right)\right)$ 3. Convolve the Ipatov symbol with CIR:$Y_{I}\left(f\right)=H\left(f\right)\cdot I_{c}\left(f\right)$ 4. CIR estimation by cross-correlation:$\hat{H\left(f\right)}=\frac{Y_{I}\left(f\right)\cdot \bar{I_{c}\left(f\right)}}{I_{c}\left(f\right)\cdot \bar{I_{c}\left(f\right)}}$   **Timstamp estimation from STS:** 5. Shift STS to carrier, complete with zero and perform FFT:$S_{c}\left(f\right)=FFT\left(e^{j2\pi f_{c}t}\cdot s\left(t\right)\right)$ 6. Generate locally replica of STS:$\hat{S_{r}\left(f\right)}=\hat{H\left(f\right)}\cdot S_{c}\left(f\right)$ 7. Simulate the received STS by convolution:$Y_{S}\left(f\right)=H\left(f\right)\cdot S_{c}\left(f\right)$ 8. Cross-correlation in the frequency domain:$R_{rs}\left(f\right)=\hat{S_{r}\left(f\right)}\cdot \bar{Y_{S}\left(f\right)}$ 9. Correlation in time domain:$r_{rs}\left(t\right)=IFFT\left(R_{rs}\left(f\right)\right)$**Output**: return $r_{rs}\left(t\right)$

**Figure 9:**
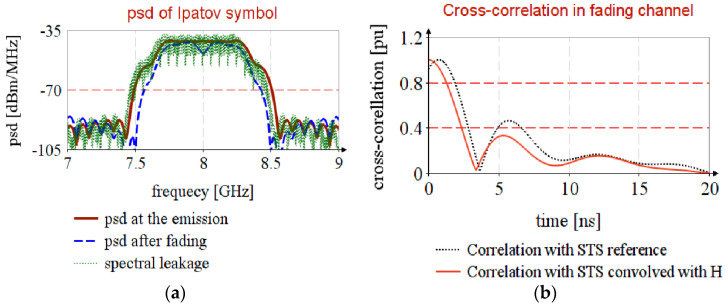
Timestamp estimation in channel with selective fading.

Figure 9a, psd at the emission, shows that Ipatov symbol has a uniform distribution over the entire bandwidth, that it follows the compliant spectral mask (Figure 2a), and that after propagation, psd after fading, it borrows CIR spectral mask.

The propagation in a channel with frequency fading leads to a small error in timestamp estimation, as shown in Figure 9b, correlation with STS reference, and this error is cancelled if the received STS is correlated with the locally generated replica, correlation with STS convolved with H.

**Figure 10:**
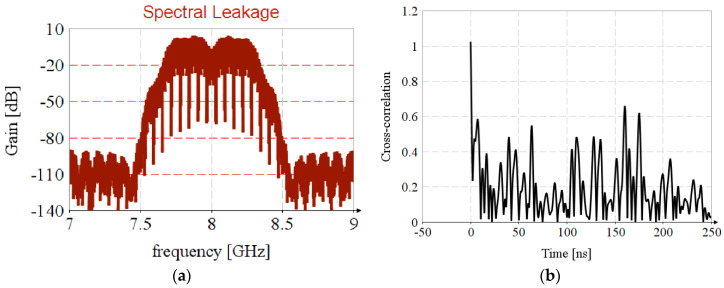
Spectral leakage in timstamp estimation: (**a**) Nonuniformity in CIR estimation; (**b**) Erroneous estimation of timestamp.

It should be highlighted that the spectral leakage in the FFT leads to the introduction of nonuniformity in the estimated CIR spectrum, Figure 10a, resulting in incorrect results regarding timestamp estimation, Figure 10b.

## 5. Results

The NESP shows the usage efficiency of the allocated bandwidth. Table 3 displays the occupied bandwidth and NESP for the pulse synthesized from entire spectral mask which is compared with previously published related works.

The uncertainty in range estimation for noisy channel with multipath propagation is computed based on Equation (4). The effective bandwidth is computed based on Equation (3). The leading lobe in Figure 7b is extracted, the samples series is completed with zero and then transformed in frequency domain. The effective bandwidth is nearly constant β=234 MHz.

The propagation loss is determined considering outdoor LOS channel with path loss exponent n=1.76,

(10) $$L=10\cdot n\cdot \log\left(\frac{d}{d_{o}}\right)$$

where $d_{0}=1\;m$ is reference distance.

The noise has psd at floor level $P_{n}-41\;\mathrm{dBm}$ and 7 GHz bandwidth.

The SNR and minimum uncertainty are depicted in Table 4.

## 6. Discussion

The synthesis of UWB impulse from a complete psd mask (Figure 2a) results in a risetime reduction, amplitude increasing, and the attenuation of side lobes relative to the impulse proposed by EiR (Table 1). Furthermore, the pulse psd fully complies with the standard spectral mask. Therefore, this impulse is advisable as a UWB reference pulse.

The random spreading of STS leads to an attenuation of lateral lobes of cross-correlation, but also leads to an increase in the probability of intra-symbol interferences. Thus, more investigations should be performed for testing these interferences in noisy channels with multipath propagation.

The cross-correlation in a noisy channel with multipath propagation displays a wide main lobe, which leads to a decrease in the accuracy of the timestamp estimation. This result seems to be due to the fact that the AES-120 sequence does not have uniform spectral distribution in the entire frequency band. The whitening of the AES sequence or of the entire STS by additional randomization could lead to the main lobe narrowing, and this is a subject of interest in our research.

## Figures and Tables

**Table 1:** The synthesis quality parameters.

Synthesis Method	Bandwidth β [MHz]	NESP [%]	Risetime [ns]	Amplitude [V]
802.15.4z reference pulse [11]	533	94.5	1.5	0.164
Pulse synthesized from entire spectral mask (Figure 2a)	535	98.5	1.2	0.187

**Table 2:** The UWB channel parameters for outdoor NLOS propagation.

Name [unit]	Symbol	Value
Path gain:		
Reference distance [m]	$d_{0}$	1
Gain at reference distance [dB]	$G_{0}$	−73
Path gain exponent	n	2.5
Frequency decaying factor	κ	0.13
**Power delay profile:**		
Expected number of clusters	$\bar{L}$	10.5
Clusters arrival rate [1/ns]	Λ	0.0243
Clusters decay time constant [ns]	Γ	104.7
Rays arrival rate [1/ns]	λ	0.223
Intracluster decay time constant [ns]	γ	9.3
First path attenuation	χ	0.65
Path increase time constant [ns]	$\gamma_{rise}$	5

**Table 3:** UWB pulse parameters.

Synthesis Method	Bandwidth β [GHz]	NESP [%]
Pulse synthesized from entire spectral mask (Figure 2a)	0.537	98.5
Gaussian with NESP maximization [6]	7.160	98.7
Linear combination of Gaussian pulses [8]	7.200	91
VC oscillator with PSO optimization [10]	1.332	NA
Gaussian with semidefinite programing [11]	12	85.5

**Table 4:** CRLB for timestamp estimation by STS in noisy channels and multipath propagation.

Distance [m]	1	10	20	40	80	160
SNR [dB]	−10.2	−27.8	−33.1	−38.4	−43.7	−49
CRLB [m]	0.1665	1.25	2.31	4.25	7.78	14.33

## Appendix A

Kolmogorov Factorization

**Algorithm A**: Kolmogorov Factorization
**Inputs**: correlation $\left\{r_{t}\right\}$, psd S(ω)
1. Inputs test:
$\left\{r_{t}\right\}$ ? Continue: Goto 3
2. Compute psd:
$S\left(\omega \right)=FFT(\{r_{t}\})$
3. Compute cepstrum coefficients:
$ReH\left(\omega \right)=\frac{1}{2}\ln S\left(\omega \right)$
4. Determine imaginary part of log filter by Hilbert transform:
REH(ω)=FFT(ReH(ω))
$Hl_{i}=\{\begin{array}{c} 0\;\;\;\;\;\;\;\;\;\;\;\;\;\;\;\;\;\;\;\;\;\;if\;\;i\equiv 0\\ -j\;REH_{i}\;\;\;\;\;\;\;if\;i>0\\ j\;REH_{i}\;\;\;\;\;\;\;\;\;\;if\;i<0 \end{array}$
ImH(ω)=IFFT(Hl)
5. Transfer function in frequency domain:
$H\left(\omega \right)=e^{ReH+j\;ImH}$
6. Transfer function in time domain of moving average type:
h=IFFT(H(ω))
7. Autoregressive function:
$a=\frac{h_{0}}{h}$
**Outputs**: return H(ω), h, a
